# Biological approaches to mitigate heavy metal pollution from battery production effluents: advances, challenges, and perspectives

**DOI:** 10.1007/s11356-025-36792-8

**Published:** 2025-09-02

**Authors:** Andrea Monroy-Licht, Walter Jose Martinez-Burgos, Júlio Cesar de Carvalho, Matheus Cavali, Adenise Lorenci Woiciechowski, Susan Grace Karp, Carlos Ricardo Soccol, Ana C. De la Parra-Guerra, Roberta Pozzan, Rosa Acevedo-Barrios

**Affiliations:** 1 Chemistry and Biology Group, Chemistry and Biology Department, Universidad del Norte, Barranquilla, 081007 Colombia; 2 Department of Bioprocess Engineering and Biotechnology, Federal University of Parana, Centro Politécnico, Curitiba, 81531-990 Parana Brazil; 3 Department of Sanitary and Environmental Engineering, Federal University of Santa Catarina, Florianópolis, 88040-370 Brazil; 4 Department of Natural and Exact Sciences, Universidad de La Costa, Barranquilla, 080002 Colombia; 5 Biodiversity Research Group of the Colombian Caribbean, Faculty of Basic Sciences, Universidad del Atlántico, Barranquilla, 081001 Atlántico Colombia; 6 Laboratory of Cell Toxicology, Department of Cell Biology, Polytechnic Center, Federal University of Paraná, Curitiba, 81531-908 PR Brazil; 7 Grupo de Estudios Químicos y Biológicos, Dirección de Ciencias Básicas, Universidad Tecnológica de Bolívar, POB 130001, Cartagena de Indias, Colombia

**Keywords:** Battery effluents, Microorganism, Bioremediation, Nanomaterials, Heavy metals, Phytoremediation

## Abstract

Battery production generates effluents containing various pollutants, predominantly heavy metals such as lead (Pb), cadmium (Cd), nickel (Ni), copper (Cu), zinc (Zn), and chromium (Cr), which represent a serious risk to human health and the environment. Given their persistence, toxicity, and mobility in ecosystems and biota, heavy metals can bioaccumulate and, in some cases, enter the food chain. With this context in mind, this review presents emerging bioremediation technologies to treat effluents from battery production, focusing on biological methods such as biosorption, phytoremediation, and the use of microorganisms. Heavy metal removal mechanisms and conventional treatments are reviewed, with emphasis on biological approaches. Biosorption emerges as the most used strategy (54.4%) across organisms from different kingdoms. In addition, existing knowledge gaps in battery industry effluent management research are identified, proposing future directions that include the integration of sustainable technologies and the use of traditional knowledge of local communities. This approach seeks not only to mitigate the environmental impact of battery production but also to promote more responsible and equitable production practices, aligned with the principles of environmental justice and sustainability.

**Graphical Abstract:**

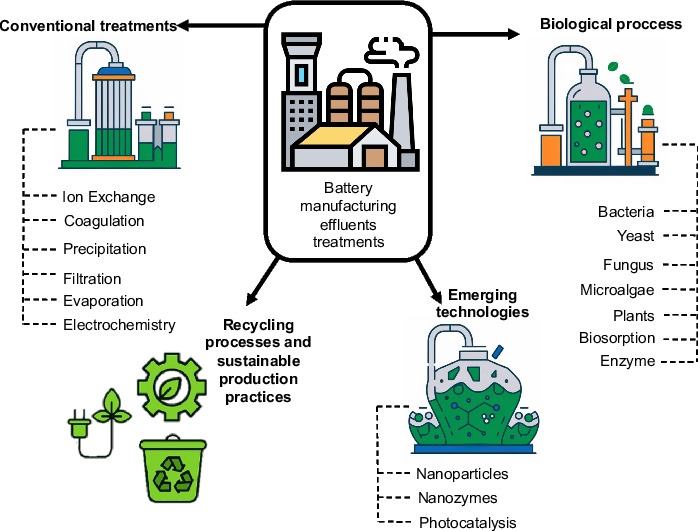

## Introduction

Large amounts of metals such as chromium (Cr), lead (Pb), zinc (Zn), nickel (Ni), and copper (Cu) are being produced worldwide every year to be used in the manufacture of different electrical and electronic devices. For instance, each year, around 20 million tons of Cu are produced on the planet and around 2.5 million tons of nickel (Su et al. 2025) and 5 million tons of lead (International-Lead-Association 2015), all of these employed in the manufacture of batteries or energy accumulators. These energy accumulators have wide applications, such as in automobiles, communication networks, renewable energy, energy storage in photovoltaic systems, energy storage devices for electrical and electronic devices, and emergency energy (Veenhuyzen et al. 2020). According to Xia et al. (2017), in 2014, around 315,000 electric vehicles were sold; in 2017, global sales reached 774,000 vehicles. For 2030, the sale of approximately 27 million electric vehicles is projected (Markets-and-Markets 2019). Therefore, significant increases in the demand for batteries in the order of gigawatt-hours (GWh) are also projected. The demand for batteries for 2025 and 2030 will increase by 3.44 and 9.3 times, respectively (Fleischmann et al. 2023).

However, the battery manufacturing industries face serious problems such as the release of traces of heavy metals (HM)—e.g., Pb, Cu, Zn, and Ni—to the environment through their effluents (Roy et al. 2024). These HMs have raised significant concern due to their ability to accumulate across various trophic levels in ecosystems, as well as their association with severe health risks in humans (Fakhri et al. 2018; Kulkarni et al. 2014) and inhibition of vital processes in plants, for example, photosynthesis (Manzoor et al. 2018; Sandeep et al. 2019). Therefore, these effluents need to be treated efficiently before being released into the environment.

Different techniques and treatment systems are used to remove heavy metals from effluents. Currently, biological systems or “bioremediation” strategies are gaining great importance due to their advantages compared with conventional treatments. This review highlights the importance of studying these effluents in the context of human health. It also provides an overview of recent advancements in bioremediation technologies for treating and reducing metals from effluents. Furthermore, the review identifies existing knowledge gaps and proposes future research directions to address these challenges.

## Methodology

The bibliographic review covered the period from 2010 to 2024, focusing on biotreatment methods for battery industry effluents. The search included keywords such as microbial remediation of effluents from the battery industry (EFBI), phytoremediation of EFBI, and enzymatic degradation of EFBI. The databases Scopus, Web of Science, and PubMed were utilized for the search. Only research and review articles published in English were considered, while books, book chapters, theses, and conference proceedings were excluded. The bibliographies of the retrieved articles were organized using the Mendeley reference manager. Additionally, data were extracted from the selected articles regarding the organisms employed in the treatment of HMs associated with effluents, removal efficiencies, and culture conditions. In some instances, older references were included, particularly for the conceptualization and description of remediation mechanisms studied in prior years. Data analysis was performed using VOSviewer (version 1.6.20).

### Results of the bibliographic search

**Fig. 1:**
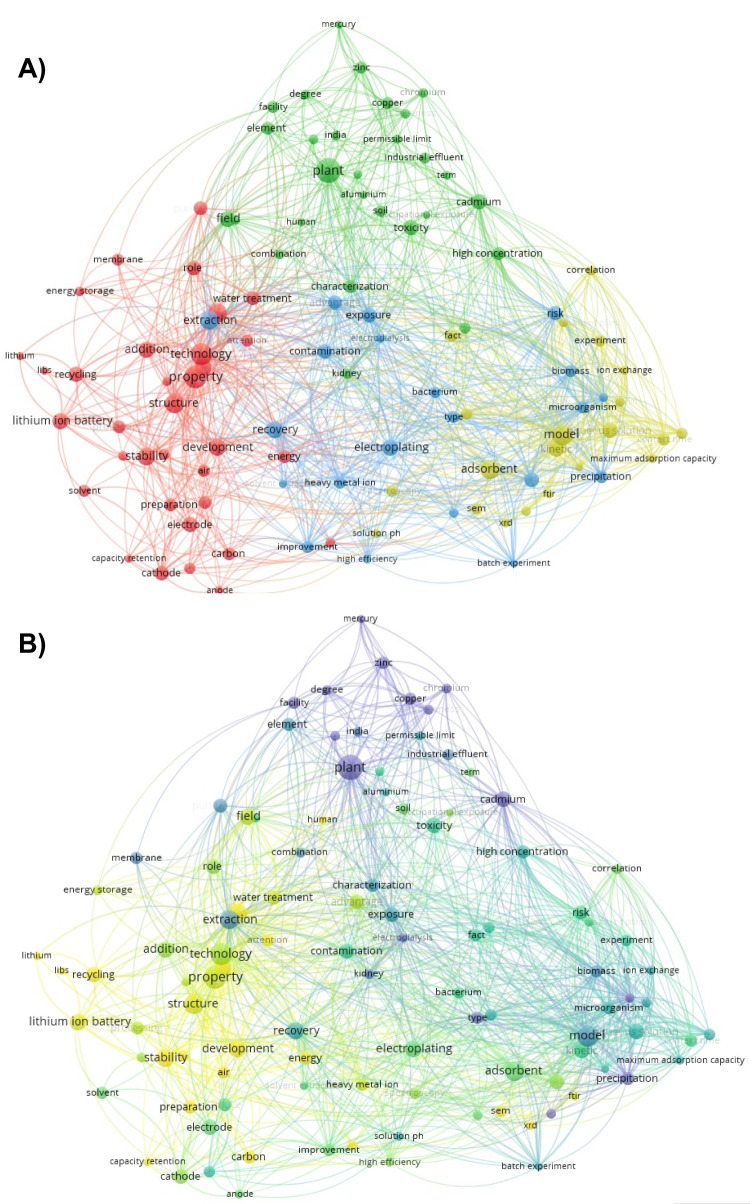
Results of the bibliographic search. **A** Cluster grouping by color according to the categorization of research topics during the reviewed period. **B** Research trends over the years, where topics from 2010 to 2014 are represented in purple, those from 2015 to 2019 in green, and topics from 2020 to 2024 in yellow. Data visualization was performed using VOSviewer (version 1.6.20)

The selection of documents included in the review was conducted following the systematic approach known as Preferred Reporting Items for Systematic Reviews and Meta-Analyses (PRISMA) (Page et al. 2021). In total, 164 articles that met the established criteria in the selected scientific databases were obtained. After removing duplicate articles, 104 documents were retained, of which 93 were research articles and 11 were review articles. The topics of the selected documents were grouped into four clusters (Fig. 1A): (i) *technologies*, this cluster includes processes such as biosorption, different matrices for biosorption, phytoremediation using macrophyte plants, and recycling processes (green color); (ii) *chemical composition of effluents*, this cluster focuses on the description of metals associated with these effluents (red color); (iii) *optimization of treatment processes*, topics in this cluster include bacterial treatments, methods such as electrodialysis, chemical and biological precipitation, and metal recovery (blue color); (iv) *modeling and process variables*, this cluster is centered on ion exchange, pH variations in both chemical and biological treatment processes, and their relationship with other contaminants present in wastewater (yellow color). Figure 1B illustrates the research trends analyzed over the years. Topics from 2010 to 2014 are represented in purple, while those from 2015 to 2019 are shown in green, and topics from 2020 to 2024 are in yellow. Emerging themes have focused on recycling, metal recovery, nanoparticles, studies on alternative biosorption materials of biological origin, modeling, the interaction of effluents with water and air, and human exposure to these effluents.

## Characteristics of effluents from battery production

Battery manufacturing processes generate significant volumes of effluents, which are classified as hazardous due to their physicochemical properties. These effluents are characterized by low pH levels and the presence of HMs, particularly Pb, with concentrations reaching up to 102 mg/L (Parvathi et al. 2007). Additionally, other HMs such as cadmium (Cd), Cr, Zn, manganese (Mn), and Ni have been detected in these effluents, further contributing to their environmental and health risks (Macchi et al. 1993; Roy et al. 2021). Table 1 summarizes the general characteristics of EFBI.

**Table 1:** Physicochemical characteristics of effluents from the battery industry (EFBI)

Physical and chemical parameters	Roy et al. (2021)	Poonam et al. (2018)	Parvathi et al. (2007)	Macchi et al. (1993)	Kumar et al. (2015)	Average
pH	2.0 ± 0.3	8.033	5.2	2.4 ± 0.39	7.1 ± 0.09	5.68
Conductivity (mS/cm)	92.2 ± 0.5	–	–	–	–	92.2
Pb (mg/L)	11.5 ± 0.2	2.393 ± 0.03	102 ± 0.03	4.02 ± 0.37	0.22 ± 0.004	24.02
Cd (mg/L)	0.02 ± 0.005	–		–	0.17 ± 0.0035	0.095
Cu (mg/L)	4.5 ± 0.6	–		0.1	2.50 ± 0.35	2.36
Fe (mg/L)	7.6 ± 0.2	–	0.28 ± 0.01	1.8 ± 0.018	–	3.22
Zn (mg/L)	28.2 ± 0.3	–	0.89 ± 0.05	0.186 ± 0.018	–	9.75
Mn (mg/L)	–	–	0.32 ± 0.02	–	–	0.32
Ni (mg/L)	–	–	0.28 ± 0.01	–	–	0.28
Cr (mg/L)	–	–	–	–	0.14 ± 0.02	0.14
NO_3_	–	–	–	25.88		25
NH_4_** (mg/L)				4.45		4.45

**Absolute value

## Human health implications of effluent contamination

The physicochemical characteristics of EFBI indicate that, even at low metal concentrations, they are highly polluting and pose significant hazards to both human health and the environment (Li et al. 2020; Xu et al. 2019). The most bioavailable and toxic form of HMs is the dissolved ionic form (Sfakianakis et al. 2015). Those pollutants are persistent, non-biodegradable (Azimi et al. 2016), and bioaccumulate in the food chain (Bora and Dutta 2019). The effects of HMs contamination are on geological, hydrological, and, finally, biological cycles (Kulkarni et al. 2014).

In general, metals present in EFBI can cause damage to living organisms at various levels. At the cellular level, heavy metals alter biomolecules, leading to modifications and loss of cell membrane permeability, the synthesis of non-functional protein-metal adducts, disruptions in the cellular redox state, the generation of free radicals and reactive oxygen species, and direct DNA damage (Haidar et al. 2023). On a macro-scale, HMs can reach humans through the consumption of food irrigated with water contaminated by EFBI, direct exposure, water consumption, or because of biomagnification processes (Khan et al. 2013). Once heavy metals enter organisms, the effects can be acute or chronic, depending on the level of contact (Saravanakumar et al. 2022). For instance, Cr^6+^ exposure is associated with cardiovascular, developmental, neurological, liver, and endocrine disorders, as well as immunological conditions and an increased risk of various cancer types in humans through inhalation and skin contact (Iyer et al. 2023). Under Cd exposure, kidney damage, liver dysfunction, skeletal and cardiovascular system effects, and visual and hearing impairments have been reported. Cd exposure is also associated with teratogenesis and mutagenesis. Additionally, it acts as an endocrine disruptor, interfering with cell signaling, human reproduction, and pregnancy development, as it can transfer from the placenta to the embryo. Furthermore, Cd has been negatively linked to reduced bone mineral density and the depletion of the body’s iron stores (Genchi et al. 2020).

In the case of Pb exposure in humans, children are particularly vulnerable due to the softness of their internal and external tissues, which increases their susceptibility to its harmful effects. Pb exposure can lead to dysfunctions in the kidneys, reproductive system, and brain. It also affects the circulatory system by inhibiting hemoglobin synthesis. In children, Pb exposure is associated with reduced IQ, learning difficulties, diarrhea, anemia, and skin allergies. As an endocrine disruptor, Pb impacts the male reproductive system, reducing sperm count and affecting fertility. Additionally, it has detrimental effects on intrauterine development (Collin et al. 2022).

**Fig. 2:**
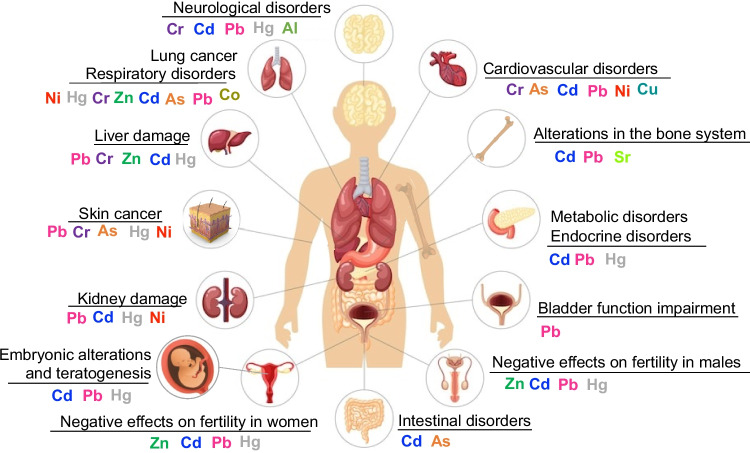
Key heavy metals of concern in public health and their associated health effects

When it comes to Hg, its target organs are the skin, lungs, liver, kidneys, and brain. With symptoms including tremors, sleeplessness, memory loss, headaches, neuromuscular effects, and cognitive and motor disorders, Hg is a strong neurotoxin that has detrimental effects on the central nervous system. Additionally, Hg causes significant disruptions in various systems; it affects the digestive and immune systems and induces renal damage, and inorganic mercury salts are corrosive to the skin, eyes, and gastrointestinal tract (Guzzi et al. 2021). Concerning Ni, it is associated with respiratory disorders, including pulmonary fibrosis. It can also cause kidney damage and has an impact on the cardiovascular system, potentially leading to coronary vasoconstriction. Ni is linked to dermatitis and genotoxic effects, which may contribute to cancer development (Begum et al. 2022). Figure 2 illustrates several HMs of interest in public health and their relationship with specific pathologies.

## Advanced treatment strategies for managing battery industry effluents

**Fig. 3:**
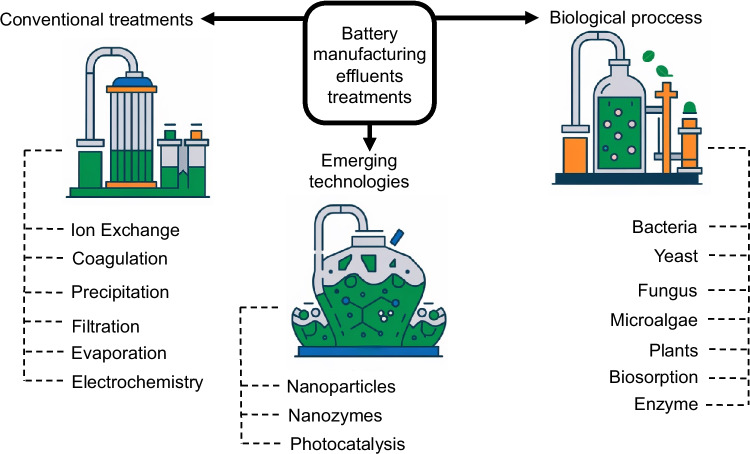
Classification of battery manufacturing effluent treatments

Given the hazardous nature of HMs and their chronic toxicity to living organisms, EFBI must undergo treatment before being discharged into the environment. The methods used can be classified into three major groups: (i) conventional or traditional methods, among which are processes of oxidation, reduction, precipitation, filtration, coagulation, electrochemistry, and evaporation (Chen et al. 2018; Gunatilake 2015); (ii) biological methods, among which are bioremediation processes with different types of organisms, microorganisms, and biosorption (Dixit et al. 2015); and (iii) new trends or emerging technologies for the treatment of effluents with heavy metals, among which are some nanomaterials and nanoenzymes (Yang et al. 2019). Figure 3 shows the general classification of treatment methods for EFBI.

### Conventional treatments

#### Chemical precipitation

One of the most used techniques to eliminate HMs is chemical precipitation (Roy et al. 2021). Generally, the reagents used are Ca(OH)_2_, Na_2_CO_3_, and Na_2_S, which bind to the metals, precipitating them (Chen et al. 2018). Among the advantages of this method, the simplicity of the operation and process control stands out. Also, it can be used in wide temperature ranges, presenting low cost and high removal efficiency. For example, Chen and colleagues achieved the removal of 99.00% of Zn and Cu by using this strategy (Chen et al. 2018). In another study, hydroxylated magnesium carbonate prepared from low-grade magnesite was evaluated as a precipitating agent to treat wastewater contaminated with heavy metals. Observing that by increasing the dose of the precipitate, the removal efficiency of metals such as VO_2_^+^, Cr^3+^, and Fe^3+^ was significantly improved, reaching values above 99.9% in only 20 min (Y. Zhang and Duan 2020). On the other hand, the use of quicklime, slaked lime, and an additional carbonation step with carbon dioxide was analyzed in wastewater from lead-acid batteries. Chemical precipitation was applied to remove sulfates and lead from the water. The results showed that both types of lime removed more than 97% of the sulfates, while the lead removal efficiency was approximately 49–53% without carbonation and increased to 68.4–69.3% with carbonation. X-ray diffraction analysis confirmed the formation of solid residues, demonstrating its potential use in the treatment of contaminated industrial waters (Vu et al. 2019).

#### Coagulation and precipitation

Coagulation and precipitation are also used to remove HMs from wastewater. These processes are mainly based on measuring the zeta potential to define the interaction between heavy metals and coagulating or flocculating agents (Gunatilake 2015). The principle of the technique is to reduce the net surface charges of the particles to facilitate their agglomeration and precipitation using flocculating agents. Among the HMs that have been removed with this technique are Pb, Cd, Cu, Zn, and Ni (Tang et al. 2014). Among the coagulating agents that have been employed are iron hydroxides and ferric sulfate; the removal efficiency ranges from 20 to 90%, depending on the type of coagulant and heavy metal to be removed (Tang et al. 2014). The minimal operating costs and convenience of using this method are among its benefits. However, it is associated with high maintenance expenses, and its efficiency tends to be highly variable. In a study, the efficiency of the coagulation-flocculation-sedimentation (CFS) process with polymeric ferric sulfate (PFS) for the removal of inorganic Sb^3+^ and Sb^5+^ species was evaluated. The results show optimum operating conditions in a pH range of 4 to 6 and demonstrated that the predominant removal mechanisms include co-precipitation by chemical bonding and, in the case of Sb(V), also electrostatic forces (Guo et al. 2018). Another investigation evaluated the use of carbamoyl benzoic acids as separation agents for the removal of Pb^2+^, Cu^2+^, and Hg^2+^ from electroplating wastewater. Association constants were determined by zeta potential, highlighting a high affinity for Pb^2+^. The compounds showed efficacy in coagulation-flocculation processes, confirmed by sedimentation kinetics and SEM–EDS analysis (Martínez-Quiroz et al. 2017). In a parallel study, the effectiveness of polyaluminum chloride (PAC) and sodium polyacrylated aluminum chloride (Magnasol 5155) in the removal of Ag from industrial wastewater by coagulation-flocculation was compared. Magnasol 5155, in combination with pH adjustment and an anionic polyelectrolyte, achieved a higher efficiency (0.004 mg/L) compared to PAC (0.027 mg/L), without increasing operating costs and favoring the generation of a sludge rich in silver (42.4 ± 3.4%), optimal for its recovery (Folens et al. 2017).

#### Electrochemical processes

Electrochemical processes at the interface of an electrical conductor and an ionic conductor have also been studied to remove HMs from EFBI. These procedures include electricity flowing through an aqueous solution that has an insoluble anode and a cathode plate (Gunatilake 2015). According to Gunatilake (2015), in electrochemical processes, HM ions are precipitated using a weak acidic catholyte or neutralized as hydroxides. Among the HM that have been removed using this process are Cu, Ni, Pb, Cd, and Zn (Choumane and Peulon 2021). The main advantages of these methods are high efficiency, operation at ambient temperature and pressures, and adjustability to effluent composition and flow. In parallel, a variation of electrodialysis has been used, involving charged ion exchange membranes that separate the metals in the solution. Nevertheless, it is crucial to remember that to achieve high efficiencies, the compounds must be ionized (Fulke et al. 2024). Additionally, the primary drawback of this technology is its high cost of maintenance (Tran et al. 2017).

For example, one study evaluated the electrochemical reduction of Cr^6+^ to Cr^3+^ using a gold electrode in acidic sodium alginate (SA) solution, followed by its removal by polymer-enhanced ultrafiltration (PEUF). The technique allowed a complete conversion of Cr^6+^ under optimal conditions (acid pH, 10 mmol/L SA), without interference by the presence of Cr^3+^, proving to be an efficient strategy for chromium removal from contaminated waters (Butter et al. 2021). In another similar case, a WO_3_/PPy-1/ACF electrode with high surface area and excellent conductivity was developed and applied in an electrochemical system to treat electroplating wastewater. This system achieved simultaneous removal of 97.8% Cu^2+^ and 80.1% citric acid in 5 h, through a synergistic reduction and oxidation mechanism, whose efficiency depended on pH, voltage, and flow rate. This technology represents an effective and low-cost alternative for the removal of heavy metals and organic acids (Sun et al. 2020). Finally, in another study, an electrochemical cell with a conductive carbon fiber fabric cathode and a platinum-coated titanium anode was designed to remove Cu^2+^ and Ni^2+^ from metal sulfate solutions, reaching efficiencies of up to 97% in 20 h. The system, which operated at 10 V and pH 6.8, showed high selectivity and electrode stability and allowed the recovery of metals in the form of hydroxides, in addition to generating useful by-products such as hydrogen and oxygen. Its versatile design makes it applicable to continuous industrial processes.

#### Ion exchange

In ion exchange processes, soluble HM ions are attracted to a solid phase. These chemical reactions are reversible, and such processes can be used in effluents with low concentrations of HMs (Gunatilake 2015). A wide variety of materials of different natures can be used, such as natural, synthetic, inorganic, cationic, anionic, and amphoteric, such as zeolites, sodium titanates, titan silicates, metal sulfides, synthetic organic resins, and inorganic three-dimensional matrix (Bashir et al. 2019). According to Azimi et al. (2016), Pb^2+^ and Cu^2+^ were removed using heat-treated zeolite. High metal removal efficiency, comparatively short working times, and waste-free operation are some of the approach benefits of this method. However, ion exchange suffers from low selectivity and is sensitive to pH fluctuations.

An example of ion exchange is a study using Dowex M4195 resin for the efficient separation of Ni and Co from leachates generated by electrochemical leaching of recycled lithium-ion batteries. This method avoided additional purification steps, obtaining concentrates with purities of 99.0% for Ni and 98.5% for Co, in addition to a separate concentrate rich in Li and Mn (Strauss et al. 2021). In another study, improved sulfonated coke was developed from *Indigofera tinctoria* carbon incorporated with synthetic phenolic resin, used as a cation exchange resin for the removal of heavy metals in wastewater. The optimized preparation, made with sulfuric acid concentrations between 10 and 50%, was characterized by FT-IR, SEM, thermogravimetric analysis (TGA), and differential thermometric analysis (DTA), showing high thermal stability and adsorption capacity (Tamizharasan et al. 2023). Furthermore, another investigation reported the synthesis and characterization of ferric hydroxide nanoparticles doped on a cation exchanger (C100-Fe) with sizes between 20 and 100 nm, evaluated for the adsorption of Pb^2+^ in industrial wastewater from lead-acid batteries. The adsorbent demonstrated superior capacity, removing Pb^2+^ up to 15,000 bed volumes (BVs) before reaching the breakpoint at 50 µg/L, far outperforming granular activated carbon (GAC), GAC impregnated with hydrated ferric hydroxide (HFO) nanoparticles, and the undoped cation exchanger. In addition, C100-Fe was regenerable with 0.5% nitric acid and showed consistent efficacy in in situ pilot tests for 30 days (Pranudta et al. 2021).

#### Membrane filtration

Filtration processes have also been employed in wastewater treatment to remove HMs. Depending on the particle size that can be retained, filtration processes can be classified as reverse osmosis (membranes are not porous), ultrafiltration (pore sizes of 1 to 100 nm), microfiltration (pore sizes of 0.05 to 10 mm), nanofiltration (separation process is based on the molecular weight cutoff), and electrodialysis (it has ion-selective exchange membranes) (Azimi et al. 2016).

These processes can reach efficiencies of up to 99.9% in removing HMs (Gunatilake 2015). Among the HMs removed using filtration processes are Ni(II) and Cu(II) (Barakat and Schmidt 2010). The high-energy usage and pressures used are two of the greatest limitations (Kang et al. 2016).

Polysulfone-nanohybrid membranes incorporating graphene oxide (GO) and hydrotalcite (HT) have been developed for the treatment of effluents from lead-acid batteries. The presence of HT prevented the agglomeration of GO, which significantly improved the stability and efficiency of the system. The membranes obtained showed good hydrophobicity, mechanical resistance, and reusability without loss of lead removal efficiency (Poolachira and Velmurugan 2023). In another study, the recovery of battery-grade lithium in the form of lithium hydroxide (LiOH) from lithium chloride (LiCl) solutions was investigated using a membrane electrolysis process. For this purpose, a double-chamber electrolytic cell with selective membranes was used, which allowed the effective separation of metallic ions, achieving a final product suitable for battery application (Srishti et al. 2025). Additionally, an investigation in which an Ar/O_2_ plasma treatment was applied to multi-walled carbon nanotubes (MWCNTs), increasing their oxygenated group content and improving their negative surface charge, dispersion, and adsorptive capacity without compromising their structural integrity, is highlighted. The functionalized MWCNTs (P-CNTs) were integrated into hollow fiber membranes, increasing their hydrophilicity and efficiency. These membranes were able to remove almost 100% of zinc in synthetic solutions and approximately 80% in real wastewater by surface complexation reactions, and membrane regenerability was observed (Ali et al. 2019).

In general, the primary drawbacks of conventional methods are the use of expensive chemical reagents and high-energy consumption, even though these methods are highly effective in removing HMs from EFBI.

### Biological treatments for EFBI

Bioremediation or biological treatments of EFBI are processes in which the metabolic capacities of living organisms—plants, microalgae, fungi, and bacteria, or their enzymes—are used for mineralizing, decreasing toxicity, or removing xenobiotic compounds (Omokhagbor Adams et al. 2015; Vidali 2001). Generally, microorganisms are isolated from those contaminated sites to take advantage of their adaptation to the pollutant (Banerjee et al. 2015). Biological mechanisms involved in bioremediation include, for example, biotransformation, by which the oxidation state of the metal changes to a less toxic, less bioavailable, and more readily recoverable state. This process involves enzyme-mediated active metabolic changes (Emenike et al. 2018). Other mechanisms include biostimulation and bioaugmentation. The first consists of stimulating the growth and metabolic activity of indigenous microorganisms present in contaminated environments by adding nutrients, cofactors, carbon, and energy sources, as well as pH and redox potential modifiers, to enhance their ability to transform, immobilize, or precipitate HMs (Barba et al. 2021). In contrast, bioaugmentation involves the introduction of specific microbial strains or exogenous microbial consortia, selected for their high efficiency in transforming or immobilizing heavy metals, with the aim of accelerating or reinforcing bioremediation processes in environments where native microbiota is insufficient (Nivetha et al. 2023). On the other hand, biosorption is a metabolically passive process by which living or dormant (dead) biomass adsorbs metal ions from aqueous solutions through physicochemical interactions with their cell surface components. This mechanism is rapid, reversible, and very effective, especially in the removal of MHs. It can involve adsorption/diffusion mechanisms through cell walls and membranes, ion exchange, and chelation with cellular compounds (Karnwal 2024). The adsorption can initiate at the cell surface, causing the synthesis of proteins and polysaccharides to be stimulated, increasing the active sites on the cell surface structure. It also involves the presence of binding groups such as imidazoles, carboxylates, sulfhydryls, amines, hydroxyls, and phosphates, some positively and some negatively charged, which allow interaction with the HMs (Ramírez Calderón et al. 2020). In the case of bioprecipitation, this is a process in which organisms induce the formation of insoluble compounds by transforming the chemical conditions of the medium (pH, redox potential) or releasing reactive metabolites such as sulfides (S^2^-), phosphates (PO_4_^3^-), or carbonates (CO_3_^2^-), which react with dissolved metal ions (such as Pb^2+^, Cd^2+^, Ni^2+^, Zn^2+^) present in battery effluents, forming solid precipitates that can be removed from the system. Among the main compounds involved are metal sulfides (e.g., PbS, CdS), metal phosphates (such as Zn_3_(PO_4_)_2_), metal carbonates (such as NiCO_3_), and metal hydroxides (such as Fe(OH)_3_), which are generated from the activity of sulfate-reducing, phosphate-reducing, or urea-producing bacteria. This mechanism allows the efficient immobilization of heavy metals, facilitating their physical separation and contributing to the decontamination of industrial waters (Sreedevi et al. 2022).

**Fig. 4:**
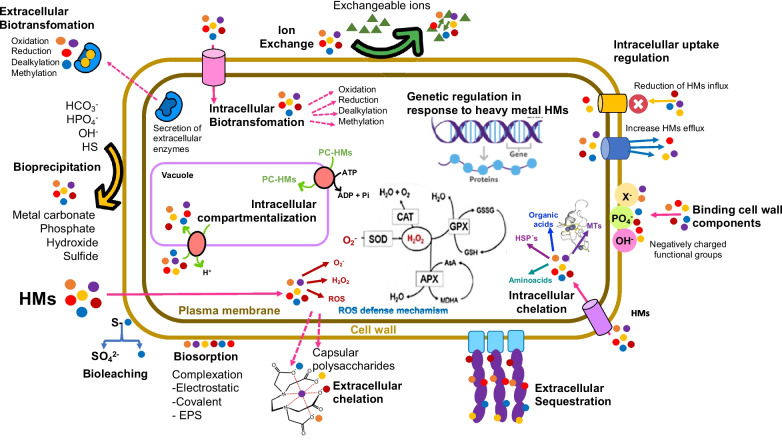
Principal mechanisms employed by organisms to eliminate or accumulate heavy metals (HMs) at the cellular level

On the other hand, in intracellular bioimmobilization, metal ions (such as Cd^2+^, Zn^2+^, or Pb^2+^) are actively transported into the cell, where they are complexed with metallothionein proteins, phytochelatins, or stored in specific organelles such as vacuoles, reducing their toxicity and preventing cell damage (Monroy-Licht 2022). On the other hand, extracellular bioimmobilization occurs outside the cell, through mechanisms such as adsorption to exopolysaccharides (EPS), the formation of biofilms, or the precipitation of metal complexes with metabolites released into the environment (such as sulfides, phosphates, or carbonates), which allows metals to be fixed in the extracellular matrix or on the cell surface (Chug et al. 2021). Figure 4 illustrates these processes.

#### Phytoremediation of EFBI

Plants possess a remarkable capacity to absorb pollutants from their surroundings and detoxify them through a variety of strategies. This capability, known as *phytoremediation*, involves the use of plants and their associated soil or water microorganisms to mitigate the concentrations or harmful effects of environmental pollutants (Greipsson 2011). This is a groundbreaking, effective, environmentally friendly, and sustainable solution that can be implemented both on-site (in situ) and off-site (ex situ). As a self-sustaining system pushed by solar energy, it is easy to operate and involves minimal expenses for setup and upkeep. This technique is well-suited for large-scale implementation and allows for the convenient removal of plant biomass. Moreover, it mitigates soil degradation and the migration of heavy metals by immobilizing them, thus lowering the potential for contaminant spread (Clemens 2001; Jacob et al. 2018).

In these organisms, inorganic contaminants and HMs are removed by phytostabilization/phytoaccumulation, phytoextraction, phytovolatilization, and rhizofiltration (Lee and Abdullah 2015; Monroy-Licht et al. 2024). Using a pretreatment procedure that guarantees metal availability is essential for EFBI phytoremediation. The properties of the metals and their speciation must also be considered. Key plant characteristics to evaluate include a rapid growth rate, high aboveground biomass production, effective tolerance mechanisms to mitigate harmful effects on plant tissues, adaptability to varying physicochemical conditions, ease of cultivation and harvesting, and measures to prevent contamination of the food chain (Li et al. 2010).

The first step in phytoextraction is the uptake. Typically, HMs and metalloids are absorbed in a manner similar to essential metals. This process involves ion transporters and complexing agents. Specialized transport proteins, such as channel proteins or H^+^-coupled carrier proteins, situated in the plasma membrane of root cells, play a crucial role in facilitating the uptake of heavy metal ions from soil or water (DalCorso et al. 2019). For instance, the ZIP family transporters are involved in metal accumulation processes and transportation of many cations (e.g., Fe, Mn, and Zn) from root to shoot (Guerinot 2000). P(1B)-ATPases are additional essential proteins that move Cu, Zn, Cd, Pb, and Co across membranes in a variety of species, involving plants (Williams and Mills 2005).

The apoplastic (passive diffusion) and symplastic (active transport against electrochemical potential gradients and concentration across the plasma membrane) routes are the two main ways whereby HMs from EFBI are taken up by roots. Metal ion carriers or complexing agents mediate the energy-dependent common absorption of HMs through the symplastic route (Yan et al. 2020). After entering the roots, HM ions may either be stored there or go mostly through xylem cells to the shoots (Rycewicz-borecki et al. 2016).

Plants also use phytostabilization as a tactic. It can happen by adsorption onto root cell walls, absorption and retention within root tissues, precipitation of HMs, or reduction of the metal valence in the rhizosphere (Gerhardt et al. 2017). Similarly, phytofiltration uses the capacity of roots (rhizofiltration), shoots (caulofiltration), or seedlings (blastofiltration) to extract HMs from contaminated EFBI (Mesjasz-Przybylowicz 2004). Table 2 provides some examples of how plants are used in metal bio-treatment and their biological mechanism.

**Table 2:** Metal accumulation in some plants and their mechanism

Pollutant	Matrix analyzed/growth medium	Organism/tissue	Maximum removal efficiency/uptake/maximum concentration in plant	Biological mechanism used	Reference
Fe*, Cu*, Cr*, Mn*, Cd*, Pb*	Wastewater from electroplating (battery, scooter, and aeronautical industry)	*Spirodela polyrhiza*	IC (µg/mL): Cu (4.83), Fe (2.63), Mn (0.36), Cr (1.55), Pb (0.18) Bioaccumulation capacity (µg/g): Cu (73.07), Fe (1070.00), Mn (126.83), Cd (12.75), Cr (128.27), Pb (20.25)		Sahu et al. 2007
		*Hydrilla verticillata*	Bioaccumulation capacity (µg/g): Cu (16.23), Fe (173.40), Mn (12.82), Cd (4.53), Cr (35.67), Pb (18.27)	Intracellular bioaccumulation	
		*Bacopa monnieri*	Bioaccumulation capacity (µg/g): Cu (32.77), Fe (293.00), Mn (86.23), Cd (7.36), Cr (111.81), Pb (16.42)		
		*Eichhornnia crassipes*	Bioaccumulation capacity (µg/g): Cu (315.50), Fe (4050.44), Mn (788.42), Cd (3.06), Cr (74.31), Pb (6.24)		
		*Ipomoea aquatica*	Bioaccumulation capacity (µg/g): Cu (29.00), Fe (966.83), Mn (127.60), Cd (11.26), Cr (26.08), Pb (15.80)		
		*Limnanthemum cristatum*	Bioaccumulation capacity (µg/g): Cu (84.94), Fe (1146.73), Mn (13.08), Cd (3.63), Cr (31.47), Pb (3.57)		
		*Marsilea minuta*	Bioaccumulation capacity (µg/g): Cu (91.83), Fe (338.83), Mn (22.27), Cd (2.36), Cr (21.38), Pb (10.65)		
Fe*, Mn*	Mixture of different types of wastewaters	*P. stratiotes* and* E. crassipes*	Lab-scale mixed wastewater ponds. Fe: 89% RE, and Mn: 74% RE	Phytostabilization, involving root accumulation and low mobility of metals to aerial tissues	Gusti Wibowo et al. 2023
Pb^2+^, Cd^2+^	Synthetic solutions	*E. crassipes* dried shoot and dried root	Removal: Pb (90%) and Cd (77%)	Biosorption via functional groups (e.g., COOH) on dried biomass surfaces	Ibrahim et al. 2012
Cu*, Zn*, As*, Cd*, Pb*	River water close to a mine outfall	*Eleocharis acicularis*	Bioaccumulation capacity (mg/kg): Cu (20,200.00), Zn (14,200.00), As (1740.00), Pb (894.00), Cd (239.00)	Hyperaccumulative phytoextraction into aerial plant tissues	Sakakibara et al. 2011
Cr^6+^, Cd^2+^	Metal-supplemented modified Murashige-Skoog medium	*Prosopis laevigata* Seedlings and dry weight	IC: Cr^6+^: 0–3.4mM and Cd^2+^: 0–2.2mM Bioaccumulation capacity (mg/kg): Cr (8176.00 seedlings), Cd (21.437 seedlings) Under 0.65 mM de Cd^2+^: Cd (5461 DW) Under 3.4 mM de Cr^6^: Cr (8090 DW)	Hyperaccumulative phytoextraction with effective root-to-shoot translocation	Buendía-González et al. 2010
Cu^2+^, Ni^2+^, Pb^2+^	Synthetic solutions	*Portulaca oleracea* Shoot	Bioaccumulation capacity (mg/kg): Cu (1940.00), Ni (1542.00), Pb (2312.00)	Phytoextraction via multi-metal hyperaccumulation in aerial tissues	Dwivedi et al. 2012
Cu*, Ni*, Cd*, Cr*, Pb*, Zn*	Household sewage and industrial waste effluents	*Basella alba*	Bioaccumulation capacity (mg/kg): Cu (7.05), Ni (2.65), Cd (0.116), Cr (2.27), Pb (2.8833), Zn (21.096)		Ratul et al. 2018
		*Cucurbita moschata*	Bioaccumulation capacity (mg/kg): Cu (14.35), Ni (4.8), Cd (0.233), Cr (1.05), Pb (5.333), Zn (12.792)		
		*Trichosanthes cucumerina*	Bioaccumulation capacity (mg/kg): Cu (11.70, Ni (3.5), Cd (0.095), Cr (0.266), Pb (2.166), Zn (17.02)	Phytostabilization with limited metal uptake and translocation	
		*Spinacia oleracea*	Bioaccumulation capacity (mg/kg): Cu (3.45), Ni (1.55), Cd (0.116), Cr (1.1), Pb (2.66), Zn (20.67)		
		*Amaranthus lividus*	Bioaccumulation capacity (mg/kg): Cu (10.316), Ni (2.1), Cd (0.283), Cr (0.95), Pb (5.50), Zn (27.226)		
Cd^2+^, Cr^6+^, As	Synthetic solutions	*Portulaca oleracea* Shoot *Portulaca tuberosa* Shoot	Bioaccumulation capacity (mg/kg): Cd (1128.00), Cr (7552.00), As (2476.00) Cd (1571.00), Cr (7957.00), As (3118.00)	Phytoextraction via shoot accumulation	Tiwari et al. 2008
Cr^6+^	Synthetic solutions	*Pteris vittata*	IC: Cr^6+^ 200 mg/L Bioaccumulation capacity (mg/kg): Cr (20,675.00)	Phytoextraction via shoot accumulation	Kalve et al. 2011
Pb^2+^, Zn^2+^, Cr^6+^, Cd^2+^, Ni^2+^	Contaminated water (aquarium experiments)	*Cyperus longus*	Pb (45.9%), Zn (44.0%), Cr (44.36%), Cd (27.5%), Ni (44.12%)	Phytoextraction via shoot accumulation influenced by pH and concentration	Soudani et al. 2024
Cr^6+^, Mn^2+^, Co^2+^, Fe^3+^, Ni^2+^, Cu^2+^, Zn^2+^, Sr^2+^, Hg^2+^, Pb^2+^	Synthetic laboratory waste solution (initial pH 1.0)	Cryogels of carboxymethyl cellulose (CMC), microparticles of *Moringa oleifera* seed husks (MS), hybrid cryogels (CMC-MS25 and CMC-MS50)	> 90% removal for all metal ions Cu^2+^ adsorption capacity: 88.2 mg/g pH adjustment to 7 with NH_4_OH for partial precipitation; fixed-bed column packed with hybrid cryogels; reusable adsorbents (5 cycles)	Adsorption via electrostatic interactions and Cu–OH chelation (confirmed by XPS)	Callisaya et al. 2024

Uptake concentration data is calculated based on the dry weight unless stated otherwise

*BCF* Bbioconcentration factor, *IC* Iinitial concentration, *DW* Ddry weight, *RE* Rremoval efficiency

* The total concentration measured in the effluent or wastewater

#### Bioremediation with bacteria

Bacteria are among the most widely utilized microorganisms in bioremediation processes, primarily due to their remarkable adaptability to diverse environmental conditions, their ability to exchange and manipulate genetic material, and their rapid growth rates (Verma and Kuila 2019). HMs, such as lead Pb, Cd, Cu, Fe, Zn, Mn, Ni, and Cr, have been effectively bioremediated using both pure bacterial strains and bacterial consortia (Kang et al. 2016; Verma and Kuila 2019). Various mechanisms have been identified to explain bacterial bioremediation of HMs, including biosorption, the production of extracellular polymeric substances (EPS), and metabolic (enzymatic) transformations. Additionally, bacteria employ subcellular sequestration as a defense mechanism against metal toxicity, converting metal ions into less harmful forms (Haferburg and Kothe 2007).

Another widely used strategy is biostimulation, which involves providing nutrients, oxygen, and other essential substances to accelerate microbial metabolic activity, thereby enhancing their ability to degrade or immobilize contaminants. For example, Fulekar et al. (2012) achieved remarkable removal efficiencies of 98.5%, 99.6%, and 100% for Cd, Cu, and Fe, respectively, from an initial concentration of 100 mg/L. This was accomplished using a microbial consortium composed of bacteria, fungi, and actinomycetes in a biostimulated process.

Various bacterial genera have been successfully utilized in HM bioremediation. Notable examples include *Achromobacter*, *Pseudomonas*, *Staphylococcus*, *Bacillus*, *Escherichia*, *Acidithiobacillus*, *Moraxella*, *Mesorhizobium*, and *Deinococcus* (Bestawy et al. 2013; Verma and Kuila 2019). For instance, *Bacillus subtilis* has been employed to remove Cd (Ivask et al. 2012), while *Escherichia coli* has demonstrated the ability to remediate multiple HMs, including Ni, cobalt (Co), Cd, and mercury (Hg) (Verma and Kuila, 20 19). Similarly, species of the *Pseudomonas* genus have been effective in removing Ni, Cd, and Hg (Chellaiah 2018). Table 3 presents some bacterial species used in the bioremediation of HMs.

**Table 3:** Bioremediation of heavy metals (HMs) using bacteria and their mechanisms

Pollutant	Matrix analyzed	Organism	Maximum removal efficiency/uptake	Biological mechanism used	Reference
Cd^2+^	Battery factory soil samples	Bacteria isolated from contaminated soil MF1, MF2, MF3, MF4, and MF5	MF1: Maximum Cd removal 85% MF2: Maximum Cd removal 75% MF3: Maximum Cd removal 29% MF4: Maximum Cd removal 33% MF5: Maximum Cd removal 27% (IC:100 mg/L)	Dual mechanism involving EPS-mediated biosorption and H_2_S-mediated cadmium biomineralization (CdS precipitation)	Chauhan et al. 2017
Cr^6+^	Synthetic effluents	*Methylococcus capsulatus*	Bioremediation of Cr^6+^ pollution over a wide range of concentrations (1.4–1000 mg/L)	Redox biotransformation mediated by methanotrophs	Hasin et al. 2010
Cr^6+^	Synthetic effluents	*Acinetobacter* sp.	Maximum Cr removal 87% (IC: 16 mg/L)	Co-metabolic reduction and biosorption coupled with ortho-pathway phenol degradation	Bhattacharya et al. 2014
Cu^2+^	Synthetic solution	*Rhodococcus* *erythropolis*	Biosorption capacity (mg/g) (IC: 100 mg/L); Cu (68.03)	Passive biosorption by non-living biomass	Baltazar et al. 2019
Pb^2+^	Synthetic effluents	*Enterobacter* sp.	Maximum Cd removal 90% (IC 1000 mg/L)	Differential biosorption and heavy metal resistance via a self-sacrifice	Jiang et al. 2020
Ni^2+^, Pb^2+^	Synthetic effluents	*Desulfovibrio desulfuricans*	(IC: 100 mg/L) Maximum removal: Ni 90.2%; Pb 98.2%	Bioprecipitation of HMs via SRB	Kim et al. 2015
Ni^2+^, Pb^2+^	Synthetic solution	*Rhodococcus* *ruber*	Biosorption capacity (mg/g) (IC: 100mg/L); Pb (42.92), Ni (6.78)	Biosorption onto bacterial biomass in solid phase extraction	Kiray et al. 2017
Ni^2+^, Cr^2+^, Cd^2+^, Pb^2+^	Battery-manufacturing waste	*Lactobacillus plantarum* MF042018	100% removal of heavy metals from the residue	Biosorption via surface adsorption following Langmuir isotherm model*	Ameen et al. 2020
Cd^2+^, Pb^2+^, Ni^2+^	Synthetic solution	*Rhodococcus* *opacus* *Rhodococcus* *rhodochrous*	Adsorption capacity (mg/g) Ni (129.34), Pb (280.11), Cd (200.8) Adsorption capacity (mg/g) Ni (154.08), Pb (401.61), Cd (215.98)	Biosorption mediated by EPS	Dobrowolski et al. 2017
Ni^2+^, Cr^6+^	Aqueous system	*Pseudomonas aeruginosa (MTCC 1688)*	Cr (26%); Ni (9%)	Biosorption mediated by EPS	Chug et al. 2021
Pb^2+^	Synthetic solution	FucoPol polysaccharide* from Enterobacter A47*	Pb^2+^ removal up to 93.9%; uptake 41.1 mg/g EPS	Biosorption mediated by EPS	Concórdio-Reis et al. 2020
Cu^2+^, Zn^2+^	Surface water (biofilter system)	*Zoogloea* sp. ZP7	Cu 84.9%; (IC 1,0 mg/L) Zn removal up to 91.7% (IC 1,0 mg/L)	Biofilm formation on iron-modified red soil carrier enhanced by microbial extracellular proteins and iron-mediated redox interactions	Zhang et al. 2024
Pb^2+^, Cu^2+^, Mn^2+^	Aqueous solution (lab-scale)	*Bacillus arachidis* (EPS-producing strain)	Pb (99.9%), Cu (99.4%), Mn (78.9%)	Biosorption via EPS containing functional groups (–OH, –COOH, amides)	Hosseini et al. 2024
Cr*, Pb*	Polluted lake water (Madiwala, Bangalore)	*Bacillus amyloliquefaciens* MEBAphL4	Cr (25.7% at pH 6); Pb (92.3% at pH 9)	Biosurfactant-mediated biosorption; lipopeptides (fengycin, iturin, surfactin) enhance removal	Biswas et al. 2024
Pb*, Hg*, Cd*	Wastewater	*Achromobacter* sp. M1	Pb (68.8 ± 0.9%); Hg (82.7 ± 1.9%); Cd (94.9 ± 1.2%) in 24 h	Biosorption + metal resistance pathways + plant growth promotion	Mahale et al. 2024
Cr*	Aqueous solution	*Bacillus nitratireducens* (live and dead cells)	86.17% removal by live cells at 100 mg/L Cr	Biosorption via absorption > adsorption; live cells showed both passive and active uptake, while dead cells only passive	Imron et al. 2024
Cd^2+^, Ni^2+^, Cr^6+^	Aqueous solution	*Bacillus xiamenensis* ISIGRM16 (from red mud)	Optimal T/pH: Cd^2+^, Ni^2+^ at 30 °C/pH 6; Cr^6+^ at 45 °C/pH 2 Cd (> 99%); Ni (85%); Cr (40%)	Biosorption via hydroxyl, carboxyl, amide groups; multilayer and monolayer adsorption	Charan et al. 2024
Cd*, Cr*	Wastewater	Modified bacterial cellulose membranes	Low-pressure filtration; high water permeability; > 90% flux recovery after 5 cleaning-filtration cycles	Filtration using functionalized bacterial cellulose with tailored ligands	Mir et al. 2024

*IC* initial concentration, *EPS* extracellular polymeric substances, *FucoPol* biodegradable, microbial exopolysaccharide, *SRB* sulfate-reducing bacteria

*Langmuir isotherm model: The fit to the Langmuir isotherm indicates that biosorption occurs on a monolayer with homogeneous adsorption sites

##### Biosorption

The positively charged HM can be sequestered in soluble and particulate form by the negatively charged cell surface through a physical–chemical process called biosorption (Gupta and Diwan 2017). Another known bioremediation mechanism is bioaccumulation, whereby HMs can potentially accumulate inside cells using cell membrane transport systems or can also participate in the cell’s biochemical processes (Abbas et al. 2014). Bioleaching processes have also been observed in the bioremediation of HMs with microorganisms (Gupta and Diwan 2017).

Microorganisms generally have two toxic heavy metal ion uptake systems (Ahemad 2019). The first process is through the chemosmotic gradient across the membrane, and the second is coupled with ATP hydrolysis. Bacteria have evolved resistance mechanisms to reduce the toxicity of ions and tolerate their absorption.

##### Extracellular polymeric substances (EPS)

Microbial macromolecules known as extracellular polymeric substances (EPS) include proteins, lipids, uronic acids, polysaccharides, nucleic acid sequences, and both organic and inorganic components (Ahemad 2019). There are two types of EPS: ropy EPS, which is a sticky slime layer, and capsular polymers (CPS), which are closely linked to the cell wall (Raj et al. 2018). Ions can immobilize metals by binding to extracellular materials, which prevents them from entering cells and causes their precipitation. It might be referred to as a bioprecipitation process because the ions readily attach to anionic functional groups as well as to the proteins, polysaccharides, and humic compounds found on the cell surface (Igiri et al. 2018). Some strains such as *Klebsiella planticola* and *Pseudomonas aeruginosa* can precipitate Cd^2+^ (Sharma et al. 2000; Wang et al. 2002), while *Vibrio harveyi* can precipitate Pb^2+^ (Mire et al. 2004).

##### Metabolic (enzyme) transformation

Bacteria also interact with HMs through their metabolic machinery, leveraging oxidation–reduction reactions to transform or detoxify these substances. Many of the bioremediation processes with different organisms are done by intra- and extracellular enzymes (Verma and Kuila 2019). The enzymes used in bioremediation processes are reductases, dehalogenases, oxygenases, cytochrome P450 mono-oxygenases, hydrolases, transferases, and oxidoreductases (Pieper et al. 2004; Rao et al. 2010). For instance, reductase enzymes, such as hydrogenases, cytochromes, and flavin reductases, are linked to the enzymatic bioremediation of Cr^6+^. These enzymes may be a component of the electron transport pathway and use chromate as the last electron acceptor (Thatoi et al. 2014).

#### Bioremediation of heavy metals using fungi and yeasts

Heterotrophic filamentous fungi can release organic acids to dissolve metals through various bioleaching processes, including acidolysis (acid–base reactions), redoxolysis, bioaccumulation, chelation, and complexation. Among the most effective fungi for bioleaching are species from the *Aspergillus* and *Penicillium* genera. These fungi are highly adaptable and resistant to environmental challenges, such as the presence of HMs (Dusengemungu et al. 2021). For instance, *Aspergillus flavus* and *Aspergillus fumigatus* strains are considered effective bioadsorbents in removing Cr^6+^ and Cd^2+^, respectively (Talukdar et al. 2020), and some yeast strains such as *Saccharomyces cerevisiae*, *Kluyveromyces marxianus*, *Candida* sp., *Schizosaccharomyces pombe* have also been used in the bioremediation of HMs (Massoud et al. 2019). Other examples of bioremediation of heavy metals with fungi and yeasts are shown in Table 4. The main advantages of using fungi in the bioremediation of HMs are their cell wall, which consists of approximately 90% biopolymers, allowing the bioadsorption of large amounts of HMs, the ease with which they bind to these contaminants, and their ability to grow in different types of substrates. Published data have shown that fungi can absorb up to 1.979 mmol (HMs)/g biomass (Das et al. 2008).

**Table 4:** Heavy metal removal by fungi and yeasts: examples, efficiencies, and mechanisms

Pollutant	Matrix analyzed	Organism	Maximum removal efficiency/uptake	Biological mechanism used	Reference
Cd^2+^, Cr^6+^	Wastewater	*Aspergillus fumigatus* + *Synechocystis* sp. PCC6803 (FMSS)	Maximum removal Cd (90.02%); Cr (80.03%)	Symbiotic system: primarily extracellular adsorption via functional groups (amino, carboxyl, aldehyde, ether); formation of precipitates (Cd(OH)_2_, Cr(OH)_3_); Cr^6+^ reduction; minor intracellular absorption	Soudani et al. 2024
Fe^2+^, Ni^2+^, Cr^6+^, Zn^2+^, As^3+^, Cu^2+^, Cd^2+^, Pb^2+^, Ag^+^, Hg^2+^	Refinery industrial wastewater	*Scedosporium apiospermum* JAZ-20	90.8–100.0%	Biosorption and biodegradation by multi-metal-tolerant fungus; process optimized	Ameen et al. 2024
Pb*, Ni*	Industrial effluent	*Aspergillus penicillioides* (metabolites used to synthesize TiO_2_ NPs)	Pb (86.9% in 4 h); Ni (25% in 2 h) Conditions: 10 µg/mL TiO_2_, pH 6, sunlight	Photocatalysis via myco-synthesized TiO_2_ nanoparticles	Vinayagam and V 2024
Cd^2+^	Stock cadmium ion solution	*Aspergillus cristatus*	Maximum Cd^2+^ removal: 88.8% (IC: 100 mg/L). enhanced uptake at pH 6 and 4 h contact	Living biomass-mediated biosorption and bioaccumulation	Hassan and El-Kassas 2012
Cu^2+^, Ni^2+^, Cd^2+^, Zn^2+^, Cr^2+^	Synthetic solution	*Beauveria bassiana*	Biosorption capacity (mg/g): Cu (4.54), Ni (4.49), Cd (4.5), Zn (3.82), Cr (3.80) (IC: 30 mg/L individual HMs)	Biosorption via cell surface functional groups and intracellular Bioaccumulation	Gola et al. 2016
Pb*, Cd*, Ni*	Industrial wastewater	*Phlebia brevispora*, *Phlebia floridensis*, and* Phanerochaete chrysosporium*	Maximum removal: Ni: 99–98% Cd: 98–97% Cd: 12–98%	Surface biosorption	Sharma et al. 2023a, b
Ni^2+^	Synthetic effluents	*Saccharomyces cerevisiae*	Maximum Ni removal: 88.8% (IC: 303 mg/L)	Passive Biosorption via cell wall functional groups	Machado et al. 2010
Cr^3+^	Tannery wastewater	*Pleurotus ostreatus*	Maximum Cr^3+^ removal: 80–90% (IC: 14.35mg/L)	Passive biosorption via functional groups in dried fungal biomass	Javaid and Bajwa 2007
Pb^2+^	Synthetic solution	*Aspergillus fumigatus*	Maximum adsorption: 85.41% (IC: 500mg/L)	Passive biosorption via cell wall functional groups	Kumar and Congeevaram 2011
Ni^2+^, Zn^2+^	Synthetic solution	*Yarrowia lipolytica*	Biosorption capacity (mg/g) Ni (30.12), Zn (44.44) (IC: 300 mg/L individual HMs)	Passive biosorption via cell wall functional groups	Wierzba 2017
Cu^2+^	Synthetic solution	*Pleurotus ostreatus* GEMB-PO1	64.56% removal at 0.5 mM Cu; 22.90% at 8 mM Cu	Biosorption and enzymatic degradation; upregulation of laccase and type II peroxidases in response to Cu—co-remediation	Gao et al. 2024
Cd*, Cu*, Pb*	Synthetic and real wastewater	*Yarrowia lipolytica* (engineered to produce sulfide)	> 90% removal of Cd, Cu, Pb ~ 550 ppm sulfide production; ambient conditions	Sulfide-mediated precipitation of metal sulfides on cell surface	Yang et al. 2024
Pb^2+^	Aqueous solution	Oxidized yeast glucan (OYG1, OYG2)	Max adsorption capacity: 100.70 mg/g (OYG1), 131.06 mg/g (OYG2) at 298 K Optimal pH 6–7	Electrostatic (ion–dipole) interaction via -COOH and -OH groups; physical and chemical adsorption	Chen et al. 2024

*IC* Iinitial concentration

* The total concentration measured in the effluent, wastewater, or water sample is used in the test

#### Bioremediation of heavy metals using Cyanobacteria and microalgae

Microalgae, present in marine environments and freshwater, are organisms with high photosynthetic efficiency and with high potential in bioremediation processes (Venkatesan et al. 2015). Considering all microorganisms mentioned in the other sections (bacteria, yeasts, and fungi), microalgae have the advantage of performing their development even under environments with nutrient stress, high salinity levels, and temperatures, as well as in the presence of HMs, which makes these microorganisms suitable for bioremediation purposes (Leong and Chang 2020). Microalgal cells need various metals in trace amounts to supply their growth (e.g., Cu, Co, Zn, Mn, Mo); nevertheless, these microorganisms also can concentrate HMs (Al-Jabri et al. 2021). Therefore, using microalgae as an alternative adsorption medium for HMs can be feasible due to its efficiency and economic aspects (Chan et al. 2014).

The processes by which microalgae absorb metals can be divided into two categories: bioaccumulation and biosorption by living and non-living cells. In the process comprising non-living cells, also named passive biosorption, the cationic metal ions are physically adsorbed by hydroxyl, carboxyl, amino, and sulfhydryl groups present on the surface of dead microalgal cells. Conversely, in the bioaccumulation or active biosorption mechanism, which regards living cells, the metal ions reach the cytoplasm by penetrating the cell membrane (Chai et al. 2021).

Developing a method for utilizing microalgae to remove HMs requires identifying and optimizing the key factors that influence the capacity of microbial species to absorb these substances. Two categories can be listed: (i) biomass factors, such as growth medium, surface properties of microalgae, and pretreatment of cells, and (ii) process factors, which comprise pH of the solution, temperature, HM concentration, and microalgae concentration, among others (Bulgariu and Gavrilescu 2015).

A recent study performed by Zada et al. (2021) reported that the microalgae assessed in their work had the potential for bioremediation applications of wastewater containing Fe^2+^. The authors also emphasized the capability of using those microalgae for bioremediation of other contaminated waters and wastewaters. Likewise, another study evaluated a simultaneous biosorption process with microalgae for a combination of HMs (Pb, Cu, Co, Cd, and Cr) to simulate an industrial application. Among the metals tested, Pb presented the best removal efficiency (higher than 90%), whereas Co, Cu, Cd, and Cr varied around 80%, 70%, 60%, and 50%, respectively (Sultana et al. 2020). The authors attributed the different removal efficiencies of HMs by active microalgae to changes in the HMs’ electronegativity.

In another work, *Didymogenes palatina XR* was cultivated under varying phosphorus concentrations to improve the capacity of removing Cd^2+^ by that strain. The microalgae removed almost 88% of Cd^2+^ from a 2 mg/L solution. After phosphorus modification, the phosphoric groups presented the predominant role in the adsorption process, increasing the capability of Cd^2+^ removal by *D. palatina XR* (Wang et al. 2021)*.* Kumar and colleagues evaluated the acclimatization of *Arthrospira platensis* by a gradual exposition to increasing HM concentrations up to 100 mg/L. The acclimatization was successful since the efficiencies obtained for the 100 mg/L HM solution were 74.6%, 68.9%, 74.1%, 52.8%, 47%, and 51.7 for Cd, Pb, Cr, Cu, Ni, and Co, respectively (Kumar et al. (2020).

A study conducted on *Desmodesmus* sp. MAS1 and *Heterochlorella* sp. MAS3, both acid-tolerant microalgae, revealed their potential for removing Fe, Mn, Cu, and Zn during growth, with subsequent application in biodiesel production. The Fe removal efficiency was approximately 80% for *Desmodesmus* sp. MAS1 and ranged from 30 to 40% for *Heterochlorella* sp. MAS3. For Mn, removal efficiencies were 37–40% for *Desmodesmus* sp. MAS1 and 32–61% for *Heterochlorella* sp. MAS3. In the case of Cu, *Desmodesmus* sp. MAS1 removed 27% of the metal, while *Heterochlorella* sp. MAS3 could remove 43%; however, this was only achieved with the lowest concentration of the metal (0.5 mg/L) because no growth was possible at higher concentrations. The best removal of Zn (68%) was reached with *Desmodesmus* sp. MAS1 in 10 mg/L Zn solution, while *Heterochlorella* sp. MAS3 was not efficient for this metal (Abinandan et al. 2019). Table 5 summarizes additional studies focused on the elimination of HMs using microalgae and Cyanobacteria.

**Table 5:** Examples of heavy metal removal by protists, Cyanobacteria*,* and microalgae: efficiency and mechanisms

Pollutant	Matrix analyzed	Organism	Maximum removal efficiency/uptake	Biological mechanism used	Reference
Pb^2+^	Synthetic multi-metal aqueous solution	*Phacus* spp.	79.2%, 2.09 mg/g	Bioaccumulation and passive biosorption via cell wall binding	Ahmad et al. 2020
Ni^2+^			66.7%, IC: 8.82 mg/g		
Al^3^⁺			64.3%, IC:16.9 mg/g		
Zn*	Mixed water samples: sewage, sea, well water	*Chlorella vulgaris*	65.0%	Biosorption, bioaccumulation, metabolic uptake	El-Sheekh et al. 2016
Cu*			56–100%		
Mn*			91.5–100%		
Ni*			51.1–100%		
Co*			32.3–59.3%		
Fe*			100%		
Cr*			21.7–66.5%		
Zn*	Mixed water samples: sewage, sea, well water	*Chlorella salina*	15.2–28.5%	Biosorption, bioaccumulation, metabolic uptake	
Cu*			90–100%		
Mn*			90–93.7%		
Ni*			82–100%		
Co*			48–100%		
Fe*			97.2–100%		
Cr*			5.1–30.6%		
Fe^2+^	Contaminated water and soil	*Chlorella fusca*	98%	Biosorption with tolerance up to 50 ppm Fe^2+^; low toxicity; bioadsorption and bioaccumulation	Zada et al. 2021
		*Ankistrodesmus braunii*	99%		
		*Scenedesmus obliquus*	97%		
		*Chlorella saccharophila*	97.5%		
		*Leptolyngbya* sp*.*	99.9%		
Cu*	Water	*Spirulina platensis*	Up to ~ 98% removal at 3 ppm Cu	Absorption of Cu by biomass; metal uptake affects growth	Budi et al. 2020
Pb^2+^	Synthetic wastewater (single and multi-metal solutions)	*Chlorella kessleri*	97.1%	Biosorption following pseudo-second order kinetics; optimized by RSM-DF and RSM-CSA modeling	Sultana et al. 2020
V^5+^	Aqueous solution with vanadium V^5+^	*Chlorella sorokiniana* and* Picochlorum oklahomensis*	Maximum removal: 25.5 mg/L; biomass yield 3.0 g/L; lipid yield 884.4 mg/L after 14 days; removal enhanced 2–2.7-folds with pH and temperature optimization	Ionic bonding with functional groups in microalgal cell walls	Tambat et al. 2023
Cr^6^⁺	Synthetic solution optimal time ~ 100 h	*Chlorella coloniales*	97.8% 97.05% 95.15% 98.6% 96.5%	Biosorption/bioaccumulation	Jaafari and Yaghmaeian 2019
Cd^2+^					
Co^2+^					
Fe^2+^					
As^5^⁺					
Pb*, Cr*, Mn*, Fe*, Co*, Ni*, Cu*, Zn*, Cd*, Ba*, B*, Al*	River water (Cooum River, Indi	*Chlorella vulgaris, Scenedesmus dimorphus*, *Phormidium* sp.	Effective removal over 15 days; algal growth confirmed by increase in chlorophyll-a and biomass	Bioadsorption and bioaccumulation using mixed algal culture	Amal Raj et al. 2024
Fe*, Zn*, Cd*, Cu*, Al*	Coal mine wastewater	Green microalgae	Fe (85%), Zn (95%), Cd (99%), Cu (100%), Al (100%) removal	Binding and absorption mechanisms	Makhanya et al. 2021
Fe*	Steel hot-rolling wastewater	*Tetradesmus obliquus*, *Chlorella sorokiniana*, *Chlorella vulgaris*, *Arthrospira platensis*, *Arthrospira maxima (spirulina)*	97.9% (Fe)	Biosorption and biodegradation	Blanco-Vieites et al. 2022
As^−3^, Co^2+^, Cu^2+^, Fe^2+^, Mn^2+^, Zn^2+^	Synthetic wastewater	*Turbinaria turbinata* (defatted algae residue)	As (100%); Co (83%); Cu (95%); Fe (97.25%); Mn (79.69%); Zn (90.15%) 2 g/L algae dose, 3 h contact time, defatted with hexane:methanol (1:1); post-lipid extraction residue used	Biosorption via algal cell wall residues	Alotaibi et al. 2024
Cu^2+^, Fe^2+^	Freshwater culture medium	*Chlorella vulgaris*, *Scenedesmus obliquus*, 50–50% mix	Effective removal 1-week incubation in 1-L flasks Cu (98.25–99.9% *C. vulgaris*), Cu (98.75–99.1% *S. obliquus*), Cu (98.61–99.9% mix), Fe (90.22–94.05% *C. vulgaris*), Fe (85.68–99.19% S*. obliquus*), Fe (91.67–97.85% mix)	Bioremediation via metal uptake and biofilm formation	Yousefi et al. 2023
Cr^6+^, Cd^2+^	Produced water from oil and gas refinery	*Dunaliella salina*	IC: 1.1 g/L Remotion: Up to 93% Cr removal in 38 min	Biosorption by microalgae powders	Ghaed et al. 2025

*IC* Iinitial concentration, *RSM-DF* Response Surface Methodology–Desirability Function, *RSM-CSA* Response Surface Methodology–Crow Search Algorithm

* The total concentration measured in the effluent, wastewater, or water sample is used in the test

**Fig. 5:**
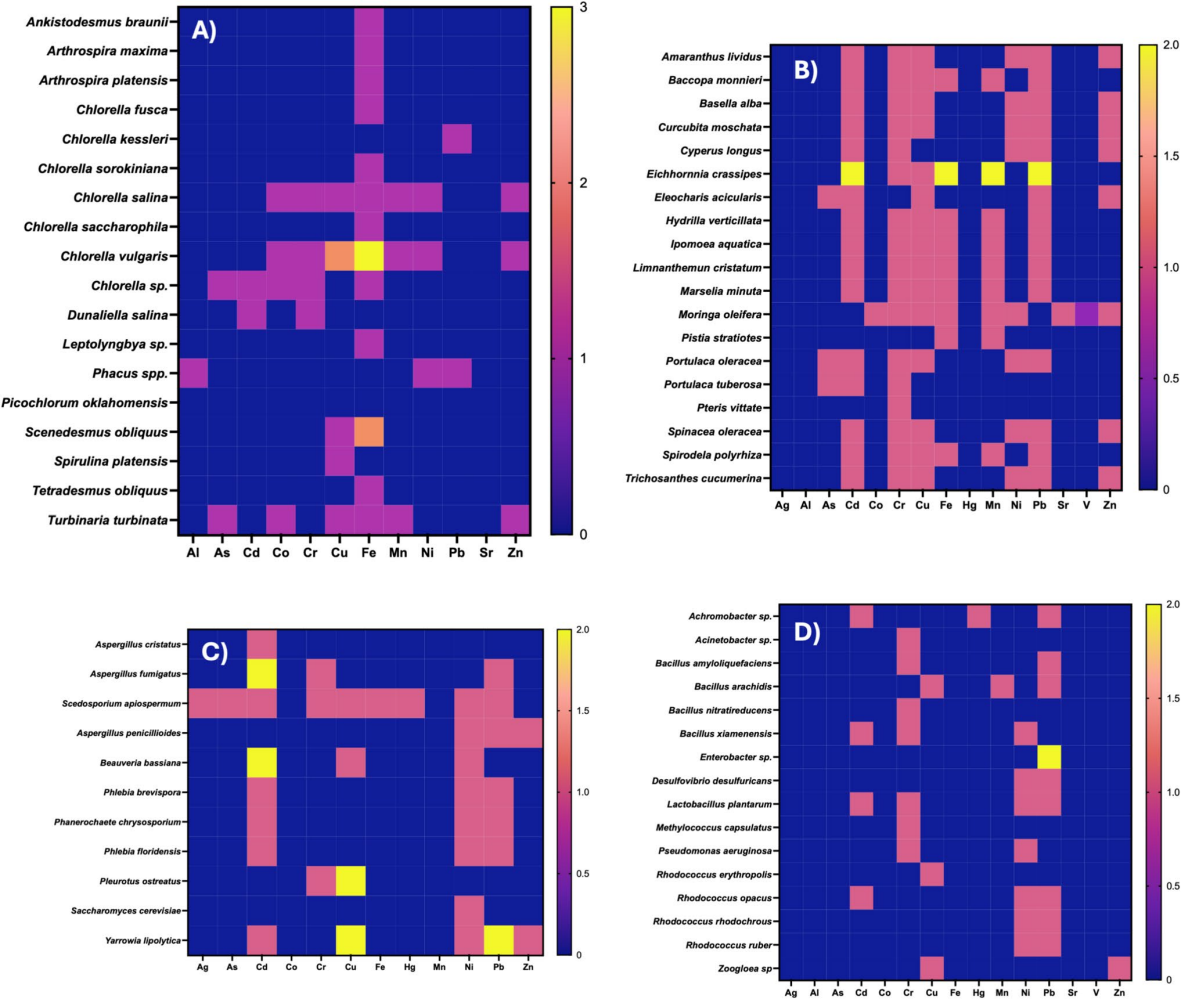
Organisms commonly used in the bioremediation of metal-contaminated effluents: **A** algae and Cyanobacteria, **B** plants, **C** filamentous fungi and yeasts, and **D** bacteria. The color scale represents the frequency of use across studies, with blue indicating fewer reports and yellow highlighting the most studied combinations of organisms and metals

According to the literature review, both Eukarya (Fig. 5A, B, and C) and Bacteria (Fig. 5D) domains are represented among the organisms used for heavy metal bioremediation. The most frequently reported group was the Plantae kingdom (48.44%), including organisms from the Chlorophyta, Polypodiophyta, and Tracheophyta phyla. The Fungi kingdom accounted for 17.19%, with species from the Ascomycota and Basidiomycota phyla. Additionally, the Chromista (1.56%; phylum Ochrophyta) and Protista (1.56%; phylum Euglenozoa) kingdoms were also represented. Within the Bacteria domain (30%), the most frequently reported phyla were Proteobacteria, Firmicutes, Actinobacteria, and Cyanobacteria.

Bioremediation strategies were classified according to their underlying mechanisms. Biosorption was the most frequent, representing 54.4% of the cases, and mainly involved functional groups of microbial or algal biomass. Bioaccumulation accounted for 8.8%, while phytoextraction and phytostabilization accounted for 7.0% and 3.5%, respectively. EPS-mediated interactions and biofilm-enhanced sorption accounted for 8.8%, and biomineralization or redox transformations each appeared in 5.3% of cases. Multi-mechanical strategies integrating sorption, degradation, or plant–microbe interactions accounted for 8.8%. These results highlight biosorption as the dominant mechanism, while highlighting the diversity of complementary biological pathways involved in metal removal.

Regarding the most frequently reported metals in the review, it can be mentioned that the highest proportion corresponds to Pb (15.79%), followed, respectively, by Cd (14, 47%), Cr (13.60%), Cu (13.16%), Fe (11.84%), Ni (11.40%), Mn (6.14%), Zn (6.14%), As (2.63%), Co (2.19%), and Hg (0.88%); in the case of Ag, Al, Sr, V, each corresponds to 0.44%

### Advantages of bioremediation in the treatment of heavy metal–contaminated effluents

Consistent with the above, bioremediation represents an environmentally sustainable and ecologically sound strategy for the remediation of contaminated environments, which is its main advantage. In addition, it is usually less expensive than other technologies (Karnwal et al. 2025). For example, it is estimated to be at least ten times cheaper than incineration and three times more affordable than physicochemical immobilization technologies (Panda et al. 2022). Traditional treatments often involve high operating costs due to the use of reagents, energy consumption, and post-treatment waste disposal (Garzón et al. 2017).

Phytoremediation has relatively low installation and maintenance costs compared to other remediation strategies, with savings of up to 5% over conventional cleanup methods without considering optimizations (H. Ali et al. 2013). For HMs, it is estimated to be 60–70% less expensive than traditional treatments (Shah 2014). In addition, bioremediation generates minimal secondary waste, and many of the organisms used (e.g., *Eichhornia crassipes*, *Bacillus* spp.) are naturally abundant or can be easily cultured at low cost (Abo-Alkasem et al. 2023).

In parallel, costs have been considered in the bio-treatment of other types of pollutants; for example, Baskaran and Byun (2024) analyzed bioremediation through artificial strategies of mixed microbial consortia for the degradation of persistent pollutants such as polycyclic aromatic hydrocarbons. The relatively low cost, the possibility of recovering value-added by-products such as biosurfactants or biofuels, and the support of tools such as artificial neural networks to optimize processes reinforce its financial viability compared to traditional methods. In addition, the analysis of costs per unit of pollutant removed allows positioning this technology in the market as a sustainable and profitable solution.

On the other hand, a study of leachate treatment based on algae and bacteria consortia, evaluated by experimental data at pilot scale in a 300 L photobioreactor, was carried out to perform an economic feasibility analysis. Initial results indicate that the operating cost per batch is approximately £170, which limits its feasibility without applying optimization strategies. The added value of this study, however, is that it delves into scenarios to reduce the costs of this bio-treatment and opens the possibility of reusing the algal biomass obtained. It proposes its use in the production of biofuels, fertilizers, food, aquaculture feed, alternative materials to plastic, and metal recovery. This offers a significant advantage over conventional methods such as reverse osmosis, which also continue to generate toxic waste. Scenario and sensitivity analysis suggests that reducing capital (CAPEX) and operating (OPEX) expenditures, along with taking advantage of economies of scale and transitioning to continuous treatment methods, can reduce total costs by as much as 85–90% (Leflay et al. 2020). This analysis, although theoretical, provides key guidelines for future research aimed at improving the financial competitiveness of microalgae-based bioremediation technologies.

In addition, some bioremediation systems, such as artificial wetlands or rhizofiltration units, require little maintenance and can operate under field conditions without major infrastructure, making them particularly advantageous in rural or resource-poor environments (Gomathi et al. 2020). Often, bioremediation can be performed directly at the site of contamination (in situ), thus eliminating transportation costs (Bhattacharya et al. 2018). These characteristics lead to a high level of public acceptance, due to their ecological approach.

The costs of bioremediation processes can also vary significantly depending on the type of environmental matrix treated, such as soils or wastewater, and site-specific conditions. For example, a study in Chile evaluated the economic performance of bioremediation of urban soils chronically contaminated with hydrocarbons using bioaugmentation strategies, biostimulation, and the combination of both, projected at industrial scale. The results showed that the cost of treatment ranged from USD 50.7 to 310.4 per cubic meter of contaminated soil. The main factors that increased costs were the use of compost in biostimulation and bacterial culture media in bioaugmentation (Orellana et al. 2022). It is important to consider within the process optimization, for example, to look for native microorganisms, which reduce the cost associated with the introduction of exogenous organisms. Being very important, the different experiences ex situ, in situ, and on site allow this type of analysis to identify critical points for process optimization and provide a useful basis for decision-making by government actors, researchers, consultants, and entrepreneurs who wish to promote a local bioremediation industry.

### Emerging technologies and new trends in the treatment of heavy metal–contaminated effluents

#### Biosorption

Solid sorbents have gained a lot of attention and use in recent years to remove HMs from EFBI. Biosorption is a physicochemical method that uses a biological matrix to capture target sorbate molecules from an aqueous solution. The sorption mechanism involves both bioabsorption and bioadsorption aspects. The assimilation (integration) of a material in one condition into another is known as absorption (e.g., liquids absorbed by solids or gases absorbed by water). In contrast, adsorption is a physical bonding process where molecules and/or ions engage with a sorbent on the surface to form a sorbent–sorbate contact (Fomina and Gadd 2014).

This is an eco-friendly, cost-effective, and efficient method of reducing the concentrations of several contaminants in various water resources to the acceptable limits suggested by regulations worldwide. In other words, it is a practical application branch of sustainable development (biotechnological approach) (Işıldar et al. 2019). Utilizing non-living biomass, biosorption is a passive absorption (metabolism-independent process) method for removing various water contaminants. The benefits of this technique include (i) recycling biomass sources (biowastes); (ii) using them in their original or modified forms to help reduce waste; (iii) having cheap operating, manufacturing, and energy costs; (iv) being widely available; and (v) having high efficiency (Abdel Maksoud et al. 2020).

Many minerals that occur naturally are seen to be promising and perfect sorbents due to their special qualities (high sorption capabilities, low cost, and abundance). Materials like chitosan, which is a by-product of N-deacetylation of chitin, have demonstrated interesting outcomes for this use; Cu, Pb, Hg, Cd, Cr, and other HMs can all be adsorbed by this naturally occurring and plentiful biopolymer (Gupta et al. 2019). Chitosan has been treated with xanthate to increase the adsorption capacity of Pb^2+^ during its removal from battery wastewater samples (Chauhan and Sankararamakrishnan 2008).

Various biomaterials have been investigated for their efficacy in removing Pb from EFBI. A study evaluated four types of eggshells for Pb removal from wastewater. The optimal dosage was 1.0 g of eggshell per 100 mL of wastewater with a contact time of 90 min at a pH of 6.0. Adsorption isotherm analysis revealed the following order of decreasing Pb removal efficiency: natural duck eggshell, natural chicken eggshell, boiled duck eggshell, and boiled chicken eggshell. The ability of eggshells to remove Pb was attributed to their physical and chemical characteristics, including a high calcium carbonate content (95–96%), porous structure, and the presence of functional groups such as carboxyl, amine, and sulfate groups (Arunlertaree et al. 2007). The capacity of raw white eggshells to absorb Co^2+^ and Li^+^ from contaminated soils was examined in a different investigation. The findings demonstrated that for incubation periods of 21 and 7 days, pH values of 5 and 4, and temperatures of 50 and 45 °C, respectively, the maximum adsorption efficiencies of Co^2+^ and Li^+^ ions were 94% and 85% (Abbas et al. 2021).

Regarding other types of bio-sorbents, Singh et al. (2014) investigated the effectivity of five different combinations of two agricultural residues as Pb^2+^ sorbents (*Arachis hypogea*, shell powder, and *Eucalyptus camaldulensis* sawdust) in lead-acid batteries wastewater through batch and column mode. The elimination of Pb^2+^ from test solutions was primarily caused by carboxyl and hydroxyl functional groups, according to the infrared spectroscopy examination. They found that the combination of 30% of *E. camaldulensis* sawdust and 70% of *A. hypogea* shell powder got the highest adsorption at pH 6 with the highest biosorption ability (*q*_max_ = 270.2 mg/g) in batch the process.

New strategies for controlling metal pollution are being developed, such as the modifications of natural polymers and their combination with nanomaterials. For example, xanthan gum (XG) is a natural compound consisting of β-(14)-d-glucopyranose units, as in cellulose, with a charged trisaccharide side chain on every alternate d-glucose residue. A modified XG polymer has been used for the removal of Pb^2+^ ions from an aqueous solution. It consisted of a nanocomposite based on nanosilica filled with modified XG polymer grafted with polyacrylamide (XG-g-PAM). This polymer could adsorb the ions from the EFBI, showing a stronger interaction of silica nanoparticles with the polymer matrix and a *q*_max_ value of 537.634 mg/g. This adsorption was spontaneous and endothermic, and the desorption studies affirmed the regenerative efficacy of loaded Pb^2+^ (Ghorai et al. 2012).

In addition, the novel use of α-cellulose fibers extracted from waste paper biomass (WP-αCFs) and magnetized with Fe_3_O_4_ NPs (M-WP-αCFs) has been tested as an adsorptive remediation of cobalt oxide nanoparticles (CoO NPs) from the water as an efficient strategy. The adsorption isotherm studies revealed a high adsorption capacity *q*_max_ (1567 mg/g). These results demonstrated the strong remediation capacity of CoO NPs and can be used to remediate several different manufactured nanomaterials (Kadam et al. 2019).

#### Treatments using nanomaterials

The use of nanomaterials in the treatment of effluents containing HMs or “nanoremediation” has recently gained relevance. Nanomaterials or nanoparticles are characterized by having dimensions at the nanoscale, usually in the range between 1 and 100 nm (Thomas et al. 2020). In addition to having unique properties due to their size, the main advantages of nanoparticles include the catalysis of reactions at high speeds due to the high area/surface ratio (Hossein et al. 2017; Kumar et al. 2017). Additionally, nanoparticles are highly specific, and several can be manufactured from ecological materials, such as biomass from microorganisms and plants. Some nanoparticles can be produced using microbes due to their high replication rate, as well as their rapid sanitization (Thomas et al. 2020).

**Fig. 6:**
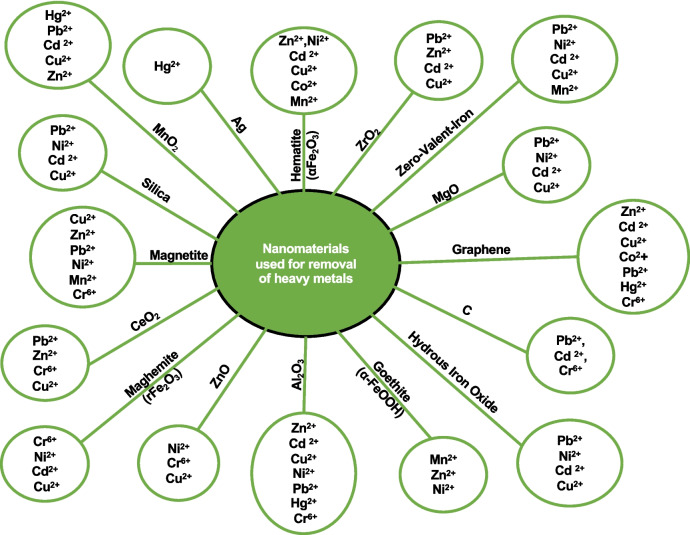
Nanoparticles employed in heavy metal bioremediation. Modified from Thangadurai et al. (2020)

Other materials, such as carbon, have also been used in the treatment of Cu, Pb, and C; mainly in the form of single- and multiple-walled nanotubes. These tubes exhibit high superiority in the treatment of effluents with HMs due to their large specific surface area and high adsorption capacity (Sudhakar et al. 2020). Another example is silica, whose nanoparticle has also been used for HM remeasurement, mainly due to its high surface area, non-toxic nature, and versatility. Cu^2+^, Pb^2+^, Cd^2+^, Ni^2+^, and Hg^2+^ are among the metals that have been removed by that material (Thomas et al. 2020). Figure 6 presents other materials used in the manufacture of nanoparticles and the metals removed by them*.*

#### Nanoenzymes

Nanoenzymes, enzyme mimetics, or next-generation enzymes are imitations of enzymes based on nanoparticles. These are defined as nanomaterials with enzyme-like activities. These compounds can catalyze reactions following a kinetic mechanism like traditional enzymes (Gao and Yan 2016). Nanoenzymes are also being used to treat different types of pollutants such as pesticides, methylene blue, and phenol (Sudhakar et al. 2020). Regarding HMs, nanoenzymes are being used to determine the presence of these pollutants (Unnikrishnan et al. 2021). The use of enzymes-nanoenzymes is gaining significant interest as a strategy to replace traditional chemical processes. Biological catalysts, particularly those engineered to enhance activity, stability, or introduce novel functions, are often more environmentally friendly than conventional chemical methods. Computational modeling tools are increasingly being utilized to optimize enzyme interactions and improve their efficiency in various remediation processes targeting contaminants of environmental concern (Bergeson and Alper 2024).

#### Bioinformatics and synthetic biology approaches for enhancing microbial remediation of battery effluents

Advances in bioinformatics tools have significantly enhanced the speed and efficiency of processes like gene sequencing, editing, and transformation, enabling the design, modeling, and integration of metabolic pathways in microbioremediation. Modern genetic technologies, utilizing state-of-the-art synthetic biology approaches, facilitate the rapid and relatively straightforward reconstruction of organism genomes. These tools are designed to address potential biophysiochemical limitations of cells, allowing them to adapt to specific environmental conditions or express desired phenotypes (Fulke et al. 2024). For instance, the microalga *Chlamydomonas reinhardtii* has been analyzed at the molecular level to enhance its photosynthetic efficiency, carbon assimilation, bioproduct synthesis, and capacity for bioremediating HMs (Sharma et al. 2023a, b). In other case, the phytochelatin synthase “PCS gene expression (SpPCS)” was genetically modified in an *E. coli* strain to remove Cd^2+^ ions (Kang et al. 2007). Similarly, the “methyltransferase gene MerR/CadC/ZntR/Pmer/PcadA/PzntA” was genetically modified in a strain of *Pseudomonas fluorescens* to remove Cd, Pb, Hg, and Zn (Bondarenko et al. 2008).

#### Photocatalysis

Photocatalysis is a catalytic oxidation method that utilizes light energy as the sole power source to generate electron–hole pairs, which are critical for breaking down contaminants. This sustainable technique has gained widespread recognition for its exceptional versatility in environmental remediation, particularly in water purification, where it effectively removes both organic and inorganic pollutants, including HMs. The concept of photocatalysis originated from studies on mimicking photosynthesis and exploring hydrogen’s potential for addressing environmental challenges.

Photocatalysts, typically semiconductor materials, possess two key energy bands: a valence band and a conduction band (Saleh et al. 2022). When exposed to light (usually visible or ultraviolet), electrons in the valence band absorb photons with energy equal to or greater than the bandgap energy, causing them to jump to the conduction band. This process creates positively charged holes in the valence band and free electrons in the conduction band (Goodarzi et al. 2023). These electron–hole pairs are highly reactive and drive redox reactions that degrade pollutants into less harmful substances, such as water, carbon dioxide, or mineralized ions.

The efficiency of photocatalysis depends on several factors, including the photocatalyst’s bandgap energy, light intensity, and the stability of the electron–hole pairs. Common photocatalysts include titanium dioxide (TiO_2_), zinc oxide (ZnO), and graphitic carbon nitride (g-C_3_N_4_), each with unique advantages and limitations. This technique has shown promising results in removing HMs like Pb, Cd, and Cr through reduction or adsorption mechanisms. Additionally, it can degrade organic pollutants, such as dyes, pesticides, and pharmaceuticals, making it a versatile tool for addressing complex environmental challenges. Despite its potential, challenges remain, including the high cost of some photocatalysts, low quantum efficiency, and the need for scalable and sustainable reactor designs, as well as the energy source being expensive, requiring high operating pressure, and not being particularly practical for small- and medium-sized enterprises (Oladimeji et al. 2024).

On the other hand, recent innovations in aqueous zinc ion batteries (AZIBs) offer promising co-benefits for the reduction of heavy metal contamination. For example, modification of zinc anodes with polydopamine (Zn@PDA) markedly improves Zn^2+^ deposition kinetics and cycling stability by mitigating dendrite and by-product formation. This improvement prolongs battery lifetime and reduces replacement frequency, which indirectly decreases the generation of metal-contaminated effluents (T. Wang et al. 2023).

In parallel, the development of functional separators, such as those modified with zirconium-based metal–organic structures (UiO-66-GF), has been shown to improve charge transport and corrosion resistance. These separators enable stable, dendrite-free zinc deposition, with Zn|UiO-66-GF-2.2|Zn cells operating reversibly for more than 1650 h and Zn|UiO-66-GF-2.2|MnO_2_ cells retaining 85% of their capacity after 1000 cycles. These advances underscore how the rational design of AZIB components can contribute to both technological sustainability and more effective management of heavy metal–contaminated effluents (Song et al. 2022).

## Gaps and limitations in the use of bioremediation for effluents generated by battery manufacturing

Battery production is a fundamental component in the transition towards clean and renewable energies, but it also generates a variety of effluents with a high potential for environmental and human health impact (Ahmad et al. 2014). One of the main current challenges lies in the analysis and treatment of these residues, exacerbated by the lack of standardization and consistency in the available data on their chemical composition, toxicity levels, and generation rates. This problem is aggravated by the limited reference information, discrepancies among studies, analytical methodologies, and reported parameters, which makes it difficult to compare results and identify optimal solutions. In many cases, the alternatives consist of the development of specific treatment systems, which are expensive and not very scalable (Yadav and Ghosh 2024). These data gaps limit the implementation of effective strategies, such as bioremediation techniques, by lacking a uniform basis that allows processes to be designed and adapted to different industrial scenarios. Therefore, it is crucial to prioritize the homogenization of information and the establishment of global standards to boost innovation and regulation in this field. In addition, challenges such as declining electrochemical performance, increasing production, and the lack of green and sustainable recycling of used batteries further complicate the picture (Wang et al. 2025). Finally, proper management of EFBI is essential not only to protect the environment but also to promote the reuse of natural resources. However, currently, a substantial number of used batteries are not recycled efficiently or sustainably, underlining the urgent need to adopt innovative and environmentally responsible solutions (Wang et al. 2025).

In addition, there is a limited number of studies with field-scale applications, making it difficult to obtain specific knowledge about the magnitude of the effects generated by battery waste in different environmental matrices and on human health. Most research focuses on limited contexts, such as landfills or industrial recycling (Gottesfeld and Pokhrel 2011). More in-depth and systematic explorations are needed to establish specific predictive models, identify environmental markers, and trace toxicity pathways according to the pollutants that generate negative effects, especially in water, soil, and air (Kumar et al. 2022). The poor functioning of battery collection centers and recycling sites, coupled with the lack of adequate processes—poorly controlled or unregulated industries—increases environmental vulnerability and aggravates the pollution caused by the components of these materials. These failures contribute, for example, to unregulated emissions of Pb into water bodies resulting from the inadequate recycling of Pb batteries (Institute for Health Metrics Evaluation 2020; Kumar et al. 2022).

A crucial aspect of recycling processes is to identify the type of battery material and assess its impact before proceeding. This includes systematic on-site testing of multiple physical and chemical parameters to better understand the associated risks and predict the effects of substances released into the natural environment. However, these practices are not always adequately implemented or, in some cases, are completely absent. Therefore, establishing a comprehensive understanding of recycled materials, together with adequate regulation and oversight, is essential to accurately assess the contamination and risks associated with battery production and handling (Jones et al. 2022; Lu et al. 2023).

The reviews conducted show significant advances in management and technologies applied in battery recycling industries. However, there are relatively few studies focused on the release of contaminants during this process and the effects that these could generate on the metabolic pathways of various organisms, including humans. This situation highlights a limited exploration of the medium- and long-term impacts that substances derived from batteries can have on ecosystems and human health (Wang et al. 2025).

Currently, there is no clear short-, medium-, or long-term mitigation pathway that effectively manages the environmental damage caused by these substances globally, nor regulations and standards to support it. Inappropriate storage of battery-derived materials poses significant risks to the environment and safety, underlining the need to promote reuse, repair, and recycling practices to minimize their negative effects (Harper et al. 2019). The metallic and non-metallic components present in batteries have the potential to become valuable resources if properly managed, but their inadequate management turns them into hazardous waste (Wang et al. 2025). This highlights the urgency of promoting scientific research that addresses different perspectives related to battery recycling. New knowledge is required to answer the multiple questions surrounding this process and its impact on industry, ecosystems, communities, and human health.

Future research challenges and recommendations should focus on developing and implementing pretreatment processes in industries to enable eco-friendly and efficient recycling of waste from various types of batteries. During pretreatment (e.g., mechanical grinding and thermal processing), numerous toxic substances are released. These include suspended particles, HMs, organic compounds, emerging contaminants, and a wide array of other toxic substances. The full composition of these compounds exhibits a high degree of chemical and physical diversity (Rada et al. 2016; Simonin and Richaume 2015). This constitutes a challenge, especially in the field of environmental toxicology and chemistry, since it is necessary to find ways for future scientific contributions to align with knowledge needs. If not adequately addressed, this problem could become an environmental time bomb, generating warning signals about imminent risks to the ecosystem. Additionally, due to the presence of components such as battery separators and plastic packaging, the pretreatment process generates the release of microplastics, new contaminants that represent an additional environmental hazard (Mercogliano et al. 2020; Wang et al. 2025).

To comprehensively address the challenges associated with battery manufacturing and the multiple factors that intensify this process, it is crucial to strengthen local capacities in underrepresented regions. These regions, although often undervalued due to their limited representation in research and policies, possess a unique natural environment that makes them strategically important (Sharma et al. 2023a, b). Underrepresented areas with significant battery production, along with other types of pollutants, represent a critical opportunity to close knowledge gaps and mitigate associated environmental impacts. However, these areas often lack specific studies assessing emissions, effluents, and waste generated by battery production, making it difficult to implement effective management and remediation strategies.

These regions are primarily home to communities with low human development indices, who face greater vulnerability due to the lack of strict regulations and adequate infrastructure. This situation aggravates pollution, negatively impacting both local ecosystems and the health of communities (De la Parra-Guerra et al. 2025). Therefore, it is essential to prioritize research that not only documents the specificities of these areas but also promotes the transfer of sustainable technologies and the design of policies adapted to their needs. This approach will allow progress towards a more equitable scenario, promoting cleaner and more responsible production practices that respect the principles of sustainability and environmental justice (Ji et al. 2015). Although current research has offered valuable methods and approaches to address this route of pollution or environmental concern, it is essential to include key areas that urgently require research in the future.

The incorporation of traditional knowledge of communities settled in these subregions is affected by distributive injustice, which generates barriers that hinder their participation in decision-making processes. This asymmetry of knowledge also negatively influences their ability to actively engage in such processes. Therefore, it is not only a matter of disseminating pre-existing information or focusing only on large industries, but of generating comprehensive knowledge that studies the environmental impacts that concern local communities, learning from their lived experience and articulating their empirical knowledge (Slattery et al. 2023).

## Conclusion

The increasing demand for batteries and the consequent generation of polluting effluents underline the urgent need for implementing sustainable and effective treatment methods. The results indicate that biological methods not only are cost-effective and easy to implement but also offer an environmentally friendly alternative to conventional physicochemical treatments.

It has been shown that EFBI have hazardous characteristics, such as low pH and high concentrations of HMs, requiring a multidisciplinary approach to their management. Despite advances in biotechnology, significant research gaps persist, especially in areas with high battery production that lack specific studies on emissions and effluents. It is essential to prioritize research in these regions, promoting the transfer of sustainable technologies and the development of policies adapted to local needs. In addition, it is crucial to address the final or intermediary destination of the remediated solids. The safe disposal, recycling, or reuse of these HMs is a critical aspect of the remediation process that requires further exploration. Developing strategies for the stabilization, recovery, or repurposing of these metals could enhance the sustainability of bioremediation technologies and contribute to a circular economy approach. Future research should focus on integrating post-remediation management practices to ensure that the entire process, from treatment to disposal, aligns with environmental and safety standards.

Furthermore, it is imperative to incorporate the traditional knowledge of affected communities to effectively address environmental challenges. Collaboration between scientists, local communities, and policymakers can facilitate the development of comprehensive strategies that address pollution while also promoting environmental justice and sustainable development.

## Data Availability

The authors confirm that all data supporting the findings of this study are included within the manuscript. Raw data can be made available by the corresponding author upon reasonable request.

## Abbreviations

Ag: Silver
As: Arsenic
BE: Biosorption efficiency
BVs: Bed volumes
Cd: Cadmium
CFS: Coagulation-flocculation-sedimentation
CPS: Capsular polysaccharides
Co: Cobalt
Cr: Chromium
Cu: Copper
DW: Dry weight
EFBI: Effluents from the battery industry
EPS: Extracellular polymeric substances
GAC: Granular activated carbon
GO: Graphene oxide
GWh: Gigawatts-hour
HBO: Hydrated ferric hydroxide
Hg: Mercury
HMs: Heavy metals
HT: Hydrotalcite
Li: Lithium
Mn: Manganese
Mo: Molybdenum
MTPs: Metal transporter proteins
MWCNTs: Multi-walled carbon nanotubes
NH_4_: Ammonium
Ni: Nickel
NO_3_: Nitrate
NRAMPs: Naturally resistant associated macrophage proteins
PAC: Polyaluminum chloride
Pb: Lead
PCS: Phytochelatin synthase
PEUF: Polymer-enhanced ultrafiltration
PFS: Polymeric ferric sulfate
P-CNT: Plasma-treated carbon nanotubes
V: Vanadium
Zn: Zinc
